# Insight into bladder cancer care: study protocol of a large nationwide prospective cohort study (BlaZIB)

**DOI:** 10.1186/s12885-020-06954-7

**Published:** 2020-05-20

**Authors:** T. M. Ripping, L. A. Kiemeney, L. M. C. van Hoogstraten, J. A. Witjes, K. K. H. Aben, on behalf of the BlaZIB study group, Joost Boormans, Theo de Reijke, Catharina A. Goossens, Sipke Helder, Maarten C. C. M. Hulshof, Geert J. L. H. van Leenders, Annemarie M. van Leliveld, Richard P. Meijer, Sasja Mulder, Ronald I. Nooter, Juus L. Noteboom, Jorg R. Oddens, Tineke J. Smilde, Guus W. J. Vanderbosch, Antoine G. van der Heijden, Michiel S. van der Heijden, Reindert J. A. van Moorselaar, Bas W. G. van Rhijn, Joep G. H. van Roermund, Bart P. Wijsman

**Affiliations:** 1 Department of Research and Development, Netherlands Comprehensive Cancer Organisation, Utrecht, the Netherlands; 2 Radboud Institute for Health Sciences, Radboud University Medical Center, Nijmegen, the Netherlands; 3 Department of Urology, Radboud University Medical Center, Nijmegen, the Netherlands

**Keywords:** Bladder cancer, Quality of care, Quality of life, Guidelines, Study protocol, Prospective cohort study

## Abstract

**Background:**

Despite the embedding of bladder cancer management in European guidelines, large variation in clinical practice exists for applied diagnostics and treatments. This variation may affect patients’ outcomes including complications, disease recurrence, progression, survival, and health-related quality of life (HRQL). Lack of detailed clinical data and HRQL data hampers a comprehensive evaluation of bladder cancer care. Through prospective data registration, this study aims to provide insight in bladder cancer care in the Netherlands and to identify barriers and modulators of optimal bladder cancer care.

**Methods:**

This study is a nationwide prospective cohort study including all patients who were newly diagnosed with high-risk non-muscle invasive bladder cancer (HR-NMIBC; Tis and/or T1, N0, M0/x) or non-metastatic muscle invasive bladder cancer (MIBC; ≥T2, N0/x-3, M0/x) in the Netherlands between November 1st 2017 and October 31st 2019. Extensive data on patient- and tumor characteristics, diagnostics, treatment and follow-up up to 2 years after diagnosis will be collected prospectively from electronic health records in the participating hospitals by data managers of the Netherlands Cancer Registry (NCR). Additionally, patients will be requested to participate in a HRQL survey shortly after diagnosis and subsequently at 6, 12 and 24 months. The HRQL survey includes six standardized questionnaires, e.g. SCQ Comorbidity score, EQ-5D-5 L, EORTC-QLQ-C30, EORTC-QLQ-BLM30, EORTC-QLQ-NMIBC24 and BCI. Variation in care and deviation from the European guidelines will be assessed through descriptive analyses and multivariable multilevel analyses. Survival analyses will be used to assess the association between variation in care and relevant outcomes such as survival.

**Discussion:**

The results of this observational study will guide modifications of clinical practice and/or adaptation of guidelines and may set the agenda for new specific research questions in the management of bladder cancer.

**Trial registration:**

Retrospectively registered in the Netherlands Trial Register. Trial identification number: NL8106. Registered on October 22nd 2019.

## Background

In the past decade, Dutch population-based studies revealed considerable variation in cancer care in general and in bladder cancer care specifically [1–3]. Part of the observed variation may be explained by case mix factors, mostly comprising patient and treatment characteristics. However, this variation may also reflect differences in hospital characteristics that affect the delivered care. For example, previous research showed that the chance of undergoing curative treatment or a cystectomy, i.e. the most delivered and recommended curative treatment in the Netherlands [4], in patients with muscle invasive bladder cancer did not only depend on patient and tumor characteristics, such as age and disease stage [5–8]. Hospital factors, like volume and type [5, 8], surgeon volume and region [6, 7] also influenced patients’ chance of undergoing a cystectomy.

Currently, only a few aspects of bladder cancer care can be evaluated: lack of detailed clinical data is hindering steps to carefully assess between-hospital practice variation. A quality of care system is required in order to improve bladder cancer care. In this system, all data necessary to detect practice variation is collected, and regular feedback to care providers and consumers is provided, all towards the goal to reduce unwanted variation in care. Furthermore, a comprehensive quality of care system is useful to identify factors that hinder or support optimal care, in order to facilitate further quality of care improvements. Until now, there is limited insight in the barriers and modulators that providers, such as treating physicians and hospitals, face in delivering optimal care. More insight in such factors is warranted to improve care for patients with bladder cancer [9].

To date, it is undecided which data, and more specifically which quality indicators based on these data, should be collected towards this goal. Multiple lists of quality indicators for bladder cancer have been designed [10–13], varying in comprehensiveness, focus, and outcomes. Although most lists focus solely on oncological outcomes, some also emphasize the need for inclusion of complementary health-related quality of life (HRQL) outcomes [10]. HRQL outcomes are especially relevant to patients and clinicians when oncological outcomes of treatment options are equal or in equilibrium, for example in the case of urinary diversion after cystectomy. Even though the lists of quality indicators vary in content, a common denominator is that they are limited in scope or mainly based on expert opinion. For example, of all quality of care indicators listed by Khare et al.*,* more than half were considered important by an expert panel, but were not supported by evidence [12].

### Contribution to the field

As described above, very limited information is available regarding (variation in) quality of bladder cancer care. There is variation in bladder cancer care, but no widely accepted evidence-based set of bladder cancer quality indicators exists to consistently and validly measure such variation. Furthermore, there is limited insight in the barriers and modulators on provider level to deliver guideline-prescribed care. Therefore, we aim to set up a prospective cohort study collecting comprehensive clinical data as well as patient-reported health-related quality of life (HRQL) data to provide insight in bladder cancer care. With these data, we will be able to reveal variation, (non-)adherence to guidelines, and factors associated with quality of care, which in potential leads to a solid foundation for evidence-based quality improvement in bladder cancer care.

### Objective

This prospective cohort study is a first step to a quality of care system for bladder cancer. It aims to gain insight in (variation of) bladder cancer care and to identify barriers and facilitating factors for optimal care.

## Methods

### Design

**Fig. 1:**
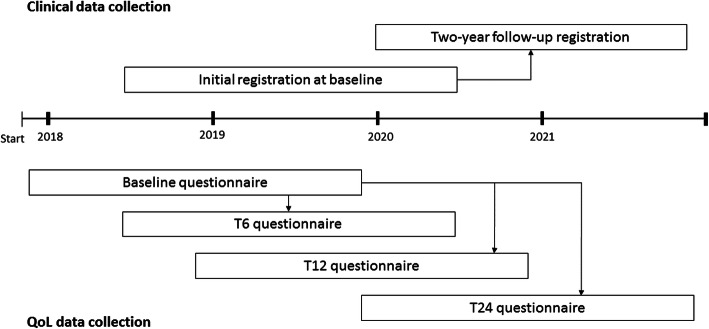
Description of clinical and HRQL data collection

This study is an ongoing nationwide prospective cohort study including Dutch bladder cancer patients diagnosed between November 1st 2017 and October 31st 2019. The study is called BlaZIB, acronym of the Dutch words ‘Blaaskanker zorg in beeld (EN: Insight into bladder cancer care), and aims to provide insight into bladder cancer care in order to improve this care. For this purpose, clinical data is collected from all eligible bladder cancer patients. Patient Reported Outcome Measures (PROMs) are collected from eligible cancer patients who are diagnosed in hospitals participating in the HRQL measures. All Dutch hospitals are invited to participate in the HRQL measures, but not all hospitals do (i.e. 53 out of 78 Dutch hospitals participated). A schematic overview of the design of the study is presented in Fig. 1.

### Characteristics of participants

Patients eligible for inclusion must be 18 years or older, have a place of residence in the Netherlands and must be newly diagnosed with high-risk NMIBC (cTis and/or cT1,N0,M0/x) or non-metastatic MIBC (cT2–4, cN0/x-3, cM0/x) in a Dutch hospital between November 1st 2017 and October 31st 2019 (about 6000 patients). All Dutch hospitals, except for one, participate in the extensive clinical data collection (*n* = 77).

Additional eligibility criteria are set for patients to participate in the HRQL measures:

1. Diagnosed in a hospital participating in the HRQL measures.
2. Able to provide informed consent.
3. Alive at time of invitation.

In case patients are deemed unable to fill out a questionnaire based on their medical record (e.g. dementia, moved to care home), they are excluded.

### Processes/methodology

Urologists are the coordinating physicians in the diagnostic phase of bladder cancer. All urologists in the Netherlands are informed about the goals of BlaZIB. One urologist (as representative of the group of urologists) of each hospital is asked for cooperation, although explicit approval is only necessary for the HRQL measurements. In the Netherlands, the National Cancer Registry (NCR) has an agreement with all individual hospitals concerning data collection of cancer patients by consulting medical files. The data collection proposed in this project falls within these established agreements. Data stored in the NCR is handled according to the Dutch law and privacy regulations. Newly diagnosed patients with bladder cancer are identified through notifications from the nationwide network and registry of histopathology and cytopathology in the Netherlands (PALGA). Data managers of the NCR select eligible patients using medical records. Patients who are eligible to participate in the HRQL measurements are invited for participation on behalf of the treating urologist. This is done by a letter which explains the general purpose of the study. In case a patient is willing to participate, the patient is asked to fill out an informed consent form and a questionnaire on HRQL. HRQL data is collected, processed and stored digitally in the Patient-Reported Outcomes Following Initial Treatment and Long-Term Evaluation of Survivorship (PROFILES) application. PROFILES is a non-profit organization that is specialized in collecting PROMs data of cancer patients [14]. Data stored in PROFILES is handled according to the Dutch law (Dutch Data Protection Act). Confidentiality and anonymity of patients is guaranteed with the assignation of a study number to each patient.

### Data collection

#### Clinical data

Clinical data are collected from medical files, including pathology and radiotherapy reports. Data managers of the NCR extract data from medical records in all hospitals, except for two hospitals in which data managers of the hospitals perform data extraction. All data are entered in the standard registration application of the NCR, which is extended to record all additional items. The registration application contains an automatic feedback system for missing data and invalid values. To ensure consistency among data managers and high quality data, a detailed coding manual is developed and manual data checks are performed regularly.

The NCR collects a standard set of data from all bladder cancer patients. These data include date of birth, date of diagnosis, topography, histology, tumor differentiation grade, clinical and pathological stage according to the most recent Tumor Nodes and Metastases (TNM) staging system of the International Union Against Cancer (UICC), initial treatment (e.g. transurethral resection of the bladder tumor (TURBT), cystectomy, radiotherapy, chemotherapy) and number of removed and positive lymph nodes. Information on hospitals involved in the diagnosis and/or treatment is available as well. Vital status is updated once every year by linkage to the Dutch Municipality Registration (GBA). The GBA contains information on all inhabitants of the Netherlands including vital status, date of death and emigration status.

Detailed information concerning diagnostic procedures and treatment as well as follow-up concerning complications, disease recurrence and progression is missing in the standard dataset of the NCR. To evaluate all relevant aspects of bladder cancer care, the current study collects extensive clinical data at baseline (i.e. collected 6 months after diagnosis) and at two-year follow-up. The additional clinical data set has been thoroughly discussed with representative medical specialists (i.e. urologists, pathologists, radiotherapists and medical oncologists) and the national bladder cancer patient society.

The additional baseline data concerns different subdomains:

- Organization and coordination of care: type of involved hospital (general, teaching, academic), multidisciplinary consultation (yes/no, involved medical disciplines, advised treatment plan, reason for deviation from treatment advise), cystectomy volume, date of first and last visit to clinical physician
- Patient characteristics: anthropometry (height, weight), family history of bladder cancer, general co-morbidity data as recorded in the Charlson Comorbidity Index, health status (World Health Organization (WHO)/Karnofsky performance score, American Society of Anesthesiologists (ASA) classification), previous operation in the abdomen, previous radiation of the pelvis.
- Tumor characteristics: multifocality, lymphovascular invasion, number of removed lymph nodes and number of removed positive lymph nodes.
- Diagnostics: date and outcome of urine cytology, date and outcome of cystoscopy.
- Imaging: date, type (Computerized Tomography (CT), Positron-emission tomography (PET), fluorodeoxyglucose (FDG)-PET/CT, Magnetic Resonance Imaging (MRI) X-ray of the thorax (X-thorax, ultrasound), region visualized, TNM.
- Blood values: creatinine, estimated glomerular filtration rate (eGFR), hemoglobin, thrombocytes, leucocytes, bilirubin, alkaline phosphatase (ALP), Aspartate transaminase (AST), Alanine transaminase (ALAT), and Lactate dehydrogenase (LDH)
- Treatment: trial participation (name trial), reason not receiving therapy, TURBT (date, type of cystoscopy, use of bladder diagram, clinical tumor size, perforation, presence of detrusor muscle in resection, visual completeness of resection), cystectomy (date, operation procedure (i.e. robot-assisted, laparoscopic, open), type of urinary diversion, operation time, peri-operative blood loss, margin status), lymph node dissection (date, extent of lymph node dissection), radiation (date, type, frequency, total dose, boost dose, elective field), bladder instillations (date, type (i.e. chemotherapy, Bacillus Calmette-Guerin (BCG)), number of instillations), chemotherapy (date, type, frequency, changes in treatment schedule, reasons for changes and use of supporting medication) and immunotherapy (date, type, reason of discontinuation)
- Outcomes: complications cystectomy (grade 2–4 according to Clavien-Dindo grading system), complications radiotherapy (grade 3–4 of the Common Toxicity Criteria for Adverse Events (CTCAE, version 5), response evaluation according to response evaluation criteria in solid tumors (RECIST), date and cause of re-admittance, survival, post-operative mortality (30-, 60-, and 90-day).

After at least 2 years of follow-up the baseline data is supplemented with data concerning complications after curative treatment, disease recurrence and progression and the applied treatment modalities.

#### PROMs

Questionnaires are administered web-based and paper-based. HRQL is measured at baseline (i.e. about 6 weeks after diagnosis) and at 6, 12 and 24 months after diagnosis using five standardized questionnaires in the Dutch translation: the Self-administered Comorbidity Questionnaire (SCQ Comorbidity score, only assessed at baseline), the EuroQol-5D-5 L (EQ-5D-5 L), the European Organization for Research and Treatment of Cancer Quality of Life Questionnaire-C30 (EORTC QLQ-C30, version 3.0), the EORTC Item Library (IL) 4-Bladder (general) and the Bladder Cancer Index (BCI) as optional questionnaire.

The SCQ Comorbidity score is developed to assess self-administered comorbidities [15]. The questionnaire includes twelve medical conditions that are frequently seen in medical practice and commonly used in comorbidity instruments such as the CCI (i.e. heart disease, high blood pressure, lung disease, diabetes, ulcer or stomach disease, kidney disease, liver disease, anemia or other blood disease, cancer, depression, arthritis, and back pain). Patients can add up to three more comorbidities themselves. For each problem, the patient can indicate the presence, severity (i.e. whether the problem is treated) and functional limitation of the problem.

The EQ-5D-5 L is a 5-item questionnaire investigating the general health of patients [16]. The questionnaire consists of two parts: a descriptive part and a visual analogue scale (VAS). The first part measures the state of health in five dimensions (i.e. mobility, self-care, usual activities, pain/discomfort and anxiety/depression) using a five-point scale defining different levels of severity. In the second part, patients rate their self-perceived health on a scale ranging from 0 (worst imaginable health state) to 100 (best imaginable health state). This questionnaire is translated in Dutch and validated.

The EORTC-QLQ-C30 was developed to assess quality of life of cancer patients in general [17]. This 30-item questionnaire contains five functional scales (physical, role, cognition, emotional, and social), three symptom scales (fatigue, pain and nausea/vomiting), and six single items assessing dyspnea, insomnia, loss of appetite, constipation, diarrhea and financial impact. Each item is scored on a 4-point scale, except for general quality of life, which is scored on a 7-point scale. After linear transformation, all scales and single item measures range from 0 to 100. A higher score on function scales and global health and quality of life scale implies a better HRQL, whereas for symptoms higher scores refer to more symptoms. This questionnaire is translated in Dutch and validated.

The EORTC-IL4-bladder (general) combines two bladder cancer specific modules of the EORTC: a Muscle Invasive Bladder Cancer module (EORTC-QLQ-BLM30) and a Non-Muscle Invasive Bladder Cancer module (EORTC-QLQ-NMIBC24). The EORTC-QLQ-BLM30 is a 30-items questionnaire that has not yet undergone factor analysis. The hypothesized scale structure of the BLM30 consists of seven scales (urinary symptom, urostomy problem, single catheter use problem, future perspective, abdominal bloating and flatulence, body image, sexual functioning) and one single item (single catheter use problem). The EORTC-QLQ-NMIBC24 is a 24-item questionnaire consisting of six scales (urinary symptoms, malaise, future worries, bloating and flatulence, sexual function, male sexual problems) and five single items (intravesical treatment issues, sexual intimacy, risk of contaminating partner, sexual enjoyment, female sexual problems). The items of the BLM30 and NMIBC24 have considerable overlap, bringing the total number of items of the EORTC-IL4-bladder (general) on 34. All items are scored on a 4-point scale and are transformed and interpreted comparable to the EORTC-QLQ-C30. Both bladder cancer specific modules of the EORTC are translated in Dutch.

The BCI is developed to assess quality of life of bladder cancer patients [18]. This 36-item questionnaire contains three scales (urinary, bowel and sexual) and two subscales (function and bother). The items are scored on a 4-point (six items) or 5-point (30 items) scale and are transformed into a scale ranging from 0 to 100. This questionnaire is validated and translated in Dutch [19].

In addition to the standardized questionnaires, several additional questions are added to obtain data on patient’s marital status, education level, employment status, smoking behavior, alcohol intake, delay to diagnosis, receiving treatment information, patient-physician decision making, disease monitoring and use of alternative medicine.

### Data management and statistical analysis

#### Statistical analysis

Descriptive analyses are performed to provide insight in the clinical and HRQL data. Clinical items are presented as mean/median/percentage (whatever is applicable), range, standard deviation and 95% Confidence Intervals (95% CI). This will be done for the total population and separately for individual institutions (e.g. hospitals, pathology labs, radiotherapy institutions) or collaborative networks of institutions. If possible, missing data will be imputed using multiple imputation procedures. Concerning the HRQL data, mean scores, standard deviations and 95% CI are presented for the different HRQL items and scores at different points in time (baseline, 6, 12 and 24 months after diagnosis). For comparison to the standard Dutch population, normative data are used when available (e.g. EORTC QLQ-C30). Analysis will be stratified and adjusted for relevant patient and tumor characteristics.

Multivariable multilevel analyses are conducted to estimate the variation between institutions and identify factors associated with this variation. The levels will be institution (e.g. volume, type of hospital), and patient/tumor characteristics (e.g. age, sex, stage, differentiation grade, social economic status, comorbidity). It should be noted that all results concerning individual hospitals will be presented in such a way that the individual hospitals will not be identifiable. Only after explicit authorization, we will present identifiable hospital-specific information. The outcome measures of the multivariable multilevel analyses are clinical measures (e.g. complications, recurrence, progression, post-operative mortality and survival) and quality of life measures. Survival analyses will be used to assess the association between variation in care and relevant outcomes such as overall, recurrence free and progression free survival. Overall and hospital-specific compliance to the European guidelines for bladder cancer (i.e. EAU guidelines) are displayed as percentages and Odds Ratios with 95% CI.

#### Power calculation

Multiple research questions will be addressed in this study aiming at providing evidence for quality indicators as surrogate measures for oncological and HRQL outcomes. The required sample size will differ depending on the specific research question as the required precision and the variation between hospitals will differ. Bladder cancer patients diagnosed in two subsequent years are included in BlaZIB. We expect to collect clinical data of about 6000 bladder cancer patients. The power calculations are based on a sample size of 6000 patients and a 95%CI. For example, if we estimate a proportion of 50% among a subgroup of 40% of all participants (e.g. patients with T1 disease, *n* = 2400), the 95% CI of the 50% will be 48.0–52.0%. When measuring variation between hospitals (*n* = 78), precision will decrease to 32–68% assuming that each hospital contributes the average number of patients. Such CIs are considered acceptable and the total number of patients included in this study will be enough to study a variety of quality indicators.

## Discussion

A quality of care system that monitors care and provides feedback to hospitals can further improve health care in countries with available and accessible care. For bladder cancer, more research is needed to define which data and quality indicators should be collected and evaluated by such quality of care system. Our study will contribute to the evaluation of potential quality indicators by providing insight in the variation of bladder cancer care and by relating this variation to relevant oncological and HRQL outcomes. In addition, our study may shed light on factors that impede or facilitate optimal quality of care. Although our study is situated in the Netherlands, we expect that our conclusions will be relevant for other countries as well. This is because the official Dutch guidelines for bladder cancer consist of the translated EAU guidelines for NMIBC and MIBC supplemented with an addendum on brachytherapy.

Our study has several strengths. First, the BlaZIB study will be the largest observational cohort study collecting clinical and HRQL data of an unselected group of bladder cancer patients to date. The BlaZIB study is incorporated in the NCR, which has nationwide coverage and receives notifications of new malignancies through linkage to the nationwide network and registry of histopathology and cytopathology in the Netherlands (PALGA). As a consequence, new bladder cancer patients can be identified quickly after diagnosis and can be invited to participate within the HRQL measures of BlaZIB, without direct involvement of medical specialists. This limits the administrative burden on physicians and prevents selection bias. Furthermore, clinical data for BlaZIB is collected via the registration system of the NRC, leading to high quality real-life data. Because of the magnitude of the BlaZIB study, in both number of participants and extent of data collection, this study can provide insight in multiple aspects of bladder cancer care and answer multiple research questions.

Another major strength of this study is that it does not stand alone, but is a first step towards continuous monitoring of quality of bladder cancer care. Based on the results of BlaZIB and other available literature, relevant quality indicators will be selected for continuous monitoring by the NCR. An advantage of nationwide monitoring through an independent institution, such as the NCR, is the guarantee of long-term continuous monitoring of quality indicators and the unburdening of medical specialists regarding registration efforts. Furthermore, members of the BlaZIB study group, who formally represent multiple medical associations, are expected to disseminate the results of BlaZIB within their medical association leading to first steps in the improvement of the quality of bladder cancer care in the Netherlands. The BlaZIB study can, therefore, be considered as the first step towards continuous monitoring of quality indicators for bladder cancer in the Netherlands.

## Conclusions

At the time of submission of this manuscript, the baseline clinical data of over 4700 patients are already registered and it is expected that this number will increase to approximately 6000 patients. Until now, 1500 patients participated in the first HRQL measurement and this number is expected to slightly increase. Based on this data, we will be able to detect variation in bladder cancer care and give insight in barriers and facilitators to optimal care.

## Data Availability

Data sharing is not applicable to this article as no datasets were generated or analyzed for the current study. One year after completion of the study, data generated in BlaZIB will be available for clinical and scientific research questions. All data requests are reviewed by the supervisory committee of the NCR for compliance with the NCR/IKNL objectives and (inter) national (privacy) regulation and legislation. For more information about these conditions, please visit: https://www.iknl.nl/en/ncr/apply-for-data

## Abbreviations

HRQL: Health-Related Quality of Life
BCI: Bladder Cancer Index
EORTC: European Organization for Research and Treatment of Cancer
QLQ: Quality Of Life Questionnaire
EAU: European Association of Urology
NMIBC: Non-Muscle Invasive Bladder Cancer
MIBC: Muscle Invasive Bladder Cancer
BCG: Bacillus Calmette-Guérin
NCR: Netherlands Cancer Registry
US: United States
TURBT: Transurethral Resection of the Bladder Tumor
GBA: Dutch Municipality Registration
BlaZIB: Insight into Bladder Cancer Care
WHO: World Health Organization
CT: Computed Tomography
PET: Positron-emission tomography
FDG: Fluorodeoxyglucose
MRI: Magnetic Resonance Imaging
ASA: American Society of Anesthesiologists
eGFR: estimated Glomerular Filtration Rate
ALP: Alkaline Phosphatase
AST: Aspartate Transaminase
ALAT: Alanine Transaminase
LDH: Lactate Dehydrogenase
PROFILES: Patient-Reported Outcomes Following Initial Treatment and Long-Term Evaluation of Survivorship
PALGA: Nationwide network and registry of histopathology and cytopathology in the Netherlands
UICC: International Union Against Cancer
